# Blind Trading: A Literature Review of Research Addressing the Welfare of Ball Pythons in the Exotic Pet Trade

**DOI:** 10.3390/ani10020193

**Published:** 2020-01-22

**Authors:** Jennah Green, Emma Coulthard, David Megson, John Norrey, Laura Norrey, Jennifer K. Rowntree, Jodie Bates, Becky Dharmpaul, Mark Auliya, Neil D’Cruze

**Affiliations:** 1 World Animal Protection 222 Gray’s Inn Rd., London WC1X 8HB, UK; BeckyDharmpaul@worldanimalprotection.org (B.D.); NeilDCruze@worldanimalprotection.org (N.D.); 2 Ecology & Environment Research Centre, Department of Natural Sciences, Manchester Metropolitan University, Manchester M1 5GB, UK; E.Coulthard@mmu.ac.uk (E.C.); D.Megson@mmu.ac.uk (D.M.); J.Norrey@mmu.ac.uk (J.N.); lauranorrey@googlemail.com (L.N.); J.Rowntree@mmu.ac.uk (J.K.R.); Jodie.Bates@stu.mmu.ac.uk (J.B.); 3 Zoological Research Museum Alexander Koenig, Department Herpetology, Adenauerallee 160, 53113 Bonn, Germany; mark.auliya@ufz.de; 4 Department of Conservation Biology, Helmholtz Centre for Environmental Research GmbH—UFZ, 04318 Leipzig, Germany; 5 Wildlife Conservation Research Unit, Department of Zoology, University of Oxford, Recanati-Kaplan Centre, Tubney House, Abingdon Road, Tubney, Abingdon OX13 5QL, UK

**Keywords:** exotic pet, *Python regius*, welfare domains, health, wildlife trade

## Abstract

**Simple Summary:**

The Ball python is a small species that is commonly kept as an exotic pet across the world. Despite huge numbers of these snakes being kept and traded in the pet industry, there is very little information available about how catching, breeding, transporting and housing them in captivity could impact their welfare. Our study reviewed the published literature for this species and found 88 relevant peer-reviewed scientific papers. Physical health was the predominant focus of research, with numerous studies reporting on disease, injury or clinical treatments. Far fewer papers focused on other aspects of Ball python wellbeing, including behaviour, nutrition, environment or mental condition. We also found that very few studies focused on wellbeing prior to pet ownership, i.e., during the early stages of the trade chain when they are caught from the wild, transported, or bred in captivity. We recommend that more research is needed to assess the impact of the exotic pet trade on this species’ welfare. In particular, research on welfare conditions during capture and transportation of wild Ball pythons, and the potential effects of captive breeding, could help reduce suffering throughout the trade.

**Abstract:**

Extensive numbers of Ball pythons are caught, bred, traded and subsequently kept in captivity across the world as part of the exotic pet industry. Despite their widespread availability as pets, relatively little is known about the potential welfare challenges affecting them. We reviewed the literature for research focused on the health and welfare of Ball pythons in the international pet trade. From a total of 88 articles returned from the search criteria, our analysis showed that very few actually focused on trade (10%) or animal welfare (17%). Instead, the majority (64%) of articles focused on veterinary science. There was a considerable bias towards physical health, with most studies neglecting the four other domains of animal welfare (behaviour, nutrition, environment and mental health). Furthermore, very few studies considered Ball pythons prior to resulting pet ownership, during wild capture and transportation or captive breeding operations. Our review demonstrates that our current understanding of welfare for Ball pythons traded as exotic pets is limited. We recommend that future research should focus on aspects of the industry that are currently overlooked, including the potential consequences of genetic selection during captive-breeding and the conditions provided for snakes prior to and during international transportation.

## 1. Introduction

Ownership of non-domesticated animals, or ‘exotic pets’, has become increasingly popular across the world [1]. The exotic pet industry is a substantial part of the global trade in wildlife products, which is worth an estimated $30.6–42.8 billion USD annually [2]. Reptiles comprise a substantial part of the live animal trade (>20%) [3] and are particularly prevalent as exotic pets in European and North American markets [3,4,5,6], for example, where around 0.9 million are kept in UK homes and 9.4 million in US homes [1,7,8]. The scale of the trade is likely to be even greater than current estimates due to incomplete record-keeping and widespread illegal activity throughout the industry [1,9]. Increasing consumer demand for novel colour and pattern strains produced by artificial breeding selection is driving industry growth for the captive breeding sector [10].

Ball pythons (*Python regius*) are one of the most common species of reptiles kept as exotic pets, dominating in trade volume [3,6]. From 1978 to 2017, between 0.9 million and 1.6 million live individuals were exported from Togo alone, 99% of which were for commercial purposes (presumably as exotic pets) [11]. The emergence of novel colour and pattern strains known as ‘morphs’ has also been a significant driver of growth in demand for captive bred Ball pythons by creating competition among breeders and owners for the most unusual and exotic characteristics [10]. Their popularity has been attributed in part to their docile nature, long lifespans and small size, deemed by some as suitable for terrariums [12,13]. Consequently, Ball pythons are often referred to as great ‘beginner’ exotic pet snakes that are relatively easy to care for in captivity [14,15].

Animal welfare refers to the wellbeing of non-human animals and is described by the American Veterinary Medical Association as the state of an animal in relation to the conditions in which it lives. There are a range of animal welfare challenges associated with the private ownership of reptiles, including Ball pythons, as exotic pets [16,17,18]. For example, Ball pythons have specific requirements regarding diet, lighting, hygiene, space, temperature and humidity [19]. Yet in a recent review involving more than 5000 individual Ball pythons in North America and Europe, D’Cruze et al. [20] found that the entities involved in this commercial enterprise were not providing housing conditions that meet the minimum welfare recommendations, either in public or privately, for periods of time that could range from several days to many years. The same study found that vendors selling Ball pythons online and at pet expositions were not providing husbandry guidance for new owners. The consequences of failing to meet these requirements or provide adequate information on how to do so can negatively impact reptile welfare, resulting in disease, injuries, stress-related behaviours [21], mental suffering and mortalities associated with poor husbandry. In addition, intense breeding selection of gene mutations to create novel morphs leads to inbreeding, resulting in genetic disorders with consequential health issues [20].

Ball pythons also face a multitude of animal welfare challenges during the trade chain prior to international export for private ownership as exotic pets. For wild caught and ranched animals, methods of capture and transportation can incite high levels of stress and physical injury [21]. Ball pythons are ranched when their eggs are taken from the wild and reared on farms. Once hatched, a proportion of individuals are returned to the wild, and the rest remain on the farm to be used commercially. Wildlife farms have been criticised for crowded or unhygienic conditions [22] that can potentially cause disease, suffering, as well as aggression or harassment from other co-occupants and competition for vital resources, such as water [23,24]. Exact mortality rates prior to and during transportation are unknown and could be significant [6,25]. Unintended harm resulting from poorly managed wild release of ranched animals (e.g., genetic pollution and disease) is also of potential concern [26].

Despite their widespread availability as exotic pets, we still know relatively little about captive reptile welfare. There is a widely acknowledged bias towards mammalian species in the scientific literature [27,28,29]. This bias extends to welfare research, where publications concerning the welfare of non-mammalian species are vastly outnumbered [30]. Herpetofauna, despite making up 46% of species richness of terrestrial vertebrates [31], are neglected in research on cognition, sentience, enrichment of captive environments and other areas of work concerning welfare prioritised in mammals and birds [28,32,33]. The lack of attention reptiles receive in comparison to other taxa has limited our understanding of their sensory abilities and functional requirements. The more we learn about reptiles, the more apparent the potential deficiencies associated with their lives in captivity become [34].

The aim of this study is to review the existing scientific literature for research focused on the health and welfare of Ball pythons in the international pet trade. We are focusing on the scientific literature, rather than associated grey literature, to examine the evidence base that knowledge throughout the industry relies on. The extensive numbers of this species that are being caught, bred, traded and kept in captivity across the world arguably warrants a thorough understanding of the potential adverse consequences associated for Ball pythons as exotic pets. In addition to searching the literature for research investigating trade, we review research pertaining to their health using search terms relating to disease and welfare. An improved understanding of the potential welfare challenges Ball pythons could experience in the exotic pet industry is imperative given the mounting physiological, neuroanatomical and behavioural evidence that reptiles are sentient beings, capable of suffering [33].

## 2. Materials and Methods

### 2.1. Literature Search

We conducted a systematic review of the scientific literature. A total of 26 search terms relating to health, welfare and trade were used (disease, pathogen, virus, viral, bacteria, bacterial, parasite, parasitic, fungus, fungal, health, welfare, exotic pet, trade, capture, transport, captive care, captive breeding, behaviour, husbandry, suffering, nutrition, diet, pain, harm, distress). Each search term was employed with the Boolean operator ‘AND’, with three additional terms (Ball python, Royal python, *Python regius*). Searches were conducted for the time period 2009–2019. Across all term combinations, 78 different searches were employed in total. This was repeated across three journal databases (PubMed(Bethesda, USA); Scopus (Amsterdam, The Netherlands); Web of Science (Philadelphia, USA). Google scholar was excluded because it returned large numbers of non-relevant literature.

### 2.2. Literature Analysis

Of the 130 papers returned from the literature search, 16 could not be sourced due to institutional access issues. A further 26 did not actually relate to Ball pythons, (only mentioned them in reference to other research) and were therefore removed from the dataset. Two papers relating to ‘Python’ software rather than animals were also removed from the returned list. This left a total of 88 papers remaining, which were included in the analysis. The literature was analysed by recording five different aspects of the content: focus, target words, use, welfare domains and pathogens.

Focus: Papers were categorised in terms of their primary focus (animal welfare, conservation, veterinary science and wildlife trade, see Appendix A), and country of origin of the study listed (where no location was given for study, the country of first author was used).

Target words: Each paper was searched for five target words, related broadly to reptile welfare: ‘welfare’, ‘suffering’, ‘pain’, ‘distress’ and ‘harm’.

Use: In reference to those papers focused specifically on wildlife trade, use of snakes in relation to the paper were categorised as ‘kill on site’, ‘capture, transport live and kill for use’ or ‘live use’.

Welfare domains: The dataset was also analysed with regards to mention of the five domains of animal welfare, a systematic assessment framework devised to assess animal’s welfare state through consideration of positive and negative experiences [35]. The experiences are split into five categories: four physical domains (environment, nutrition, physical health and behaviour) and one mental domain. Papers were recorded if they mentioned food and water deprivation, environmental challenge or discomfort, disease or injury, behavioural restriction or anxiety and stress.

Pathogens: Any mention of ‘bacteria’, ‘fungi’, ‘parasite’, ‘protozoa’ or ‘virus’ were noted as a result of searching the document. All disorders, diseases or conditions were recorded in relation to Ball pythons, with a list of specific named pathogens and parasites compiled. In addition, any recommendations made by the authors were collated.

### 2.3. Statistical Analysis

All analysis was carried out in R version 3.6.1 (R Core Development Team, 2019). Chi-square goodness of fit tests were used to investigate the distribution of papers published across research category and across locations. Results were recorded as the percentage of papers.

## 3. Results

Our results are derived from a literature analysis of 88 relevant peer-reviewed scientific papers, returned from our search criteria.

**Figure 1:**
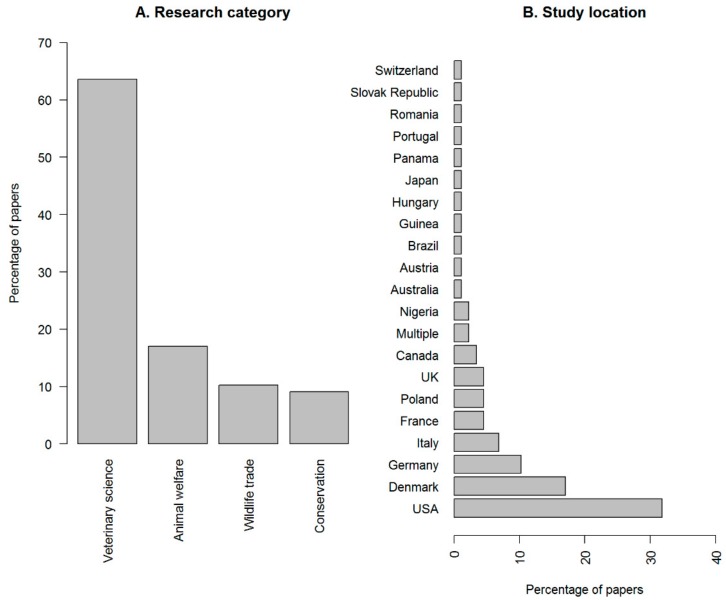
Percentage of papers per research focus category (**A**), and by location of study (**B**). Total number of papers included *n* = 88.

Focus: Figure 1A shows the percentage of papers on Ball pythons in each of the assigned primary research focus categories. There was a significantly uneven split across these categories (χ^2^ = 71.36, df = 3, *p* < 0.001), with ‘veterinary science’ (64%) being the most common focus followed by ‘animal welfare’ (17%) (Figure 1A). The location of study was also not evenly distributed (χ^2^ = 198.84, df = 20, *p* < 0.001), with the USA having the largest percentage of papers (32%), followed by Denmark, Germany, Italy, France and Poland (Figure 1B). None of the studies originated in West Africa, where Ball pythons are the most common legally exported species.

Target words: Our target word searches of the 88 papers found that 4% mentioned the word “welfare” (*n* = 4), only 1% mentioned “suffering” (*n* = 1) and 12% mentioned one of the following stress related terms: “pain”, “distress” or “harm” (*n* = 14).

Use: Details of Ball Python use in the studies concerned showed that no papers relating to wildlife trade indicated snakes were killed on sight, or indicated snakes were captured, transported and then killed for use and 77% referred specifically to live use (*n* = 55). The other 23% of papers did not specify use.

**Figure 2:**
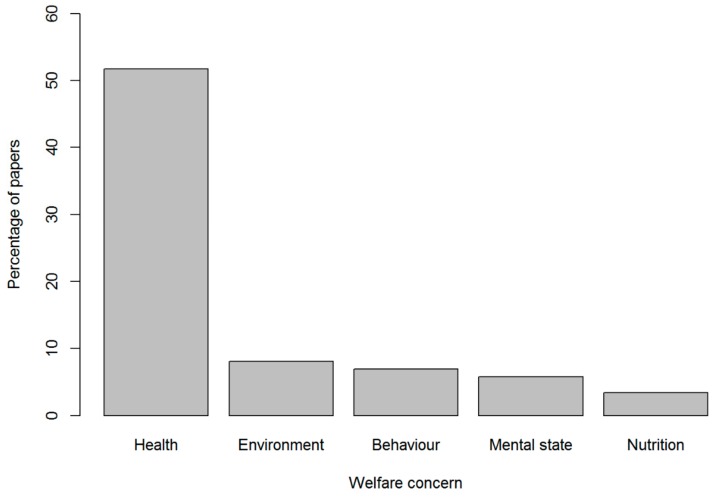
Percentage of papers that identified each of the five welfare domains.

Welfare domains: Figure 2 shows the percentage of papers that identified each of the five welfare domains. Of the 88 papers, 51% (*n* = 45) considered negative aspects of the ‘health’ domain, citing disease or injury. Within this domain, the majority of issues raised were physical illnesses (diseases, parasites, etc.), rather than behavioural issues or injury (burns, bites, etc.) (Appendix A; Appendix C Tables S1–S6). Far fewer papers mentioned the four remaining domains. Only 8% (*n* = 7) of the studies addressed the ‘environment’ domain, citing challenges and discomfort arising from the animal’s surroundings, such as the effects of inappropriate temperature and humidity. Another 7% (*n* = 6) addressed negative aspects of the ‘behaviour’ domain, concerned with abnormal behaviours such as open mouth breathing, head tremors and lethargy. Negative experiences in the ‘mental state’ domain, such as anxiety, fear and distress were mentioned in 6% (*n* = 5) of the studies. Finally, ‘nutrition’ was the least cited domain, where only 3% (*n* = 3) of the papers referred to deprivation of food and/or water, and incorrect nutrition from an inadequate diet.

**Figure 3:**
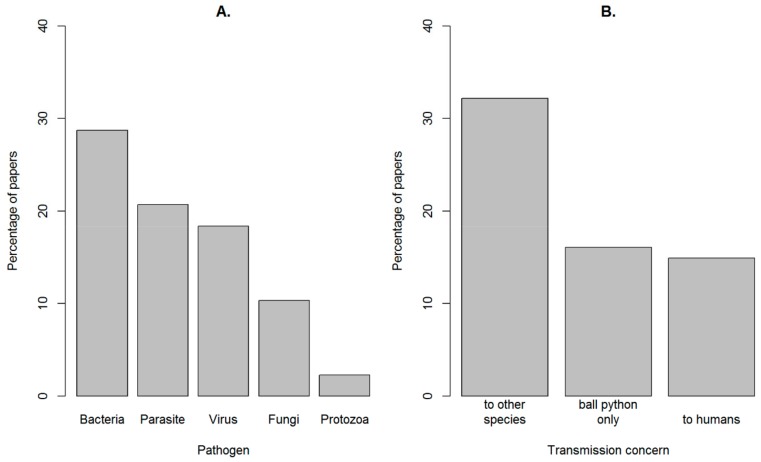
Percentage of papers that mentioned different pathogen types (**A**) and the different transmission concerns (**B**). A full list of the specific pathogen species (or taxa) is shown in Appendix C.

Pathogens: The majority of the pathogens reported were bacteria and parasites, with fewer instances of protozoa, fungi and viruses (Figure 3; Appendix C). Details of the specific health and behavioural issues related to Ball python welfare indicated by the papers are provided in Appendix B and Appendix C (with associated definitions provided in Tables S1 and S2).

## 4. Discussion

Our study provides the most comprehensive review of published literature addressing the welfare of Ball pythons in the exotic pet trade chain, to date. A total of 88 articles were returned from our search criteria, although very few specifically addressed trade (10%) or animal welfare (17%). Rather, the vast majority of articles were focused on veterinary science, describing over 100 clinical symptoms and nearly 150 underlying pathogens (Appendix B; Tables S2, S6), many of which were artificially imposed on the snakes during controlled experiments. In the context of the five domains of animal welfare, our analysis showed a considerable preference towards physical health. Around two thirds of the studies mentioned disease and injury. In contrast, fewer than 10% of published literature referred to the snake’s environment, nutrition, behaviour or mental wellbeing. Furthermore, none of the studies were conducted in, or referred to welfare and health aspects in West Africa, where Ball pythons are the most numerically exported species for use as pets listed on CITES.

### 4.1. Animal Welfare Domains

The relatively extensive coverage of the physical condition and associated clinical signs observed in captive Ball pythons could benefit their welfare as it can help enable appropriate veterinary attention and changes to husbandry practices when necessary. However, focusing the majority of research in one area while neglecting other key domains of animal welfare could have negative implications for Ball pythons. For example, only six studies (7%) looked at the snake’s behaviour, even though behavioural change in reptiles can often serve as the primary indicator of disturbance, injury or disease [24]. Behavioural assessments may also offer the advantage of detecting subclinical or psychological conditions, such as under stimulation from their environment, that may not be revealed through physiological measurement [24]. Informed behavioural assessment of reptiles could therefore be a valuable and non-invasive means of evaluating welfare [36,37]. Thus far, this appears to have been underutilised in the assessment of Ball pythons in the exotic pet trade chain.

Similarly, many negative health and welfare experiences are a consequence of poor nutrition or an inadequate captive environment [34]. A poor diet can cause a range of diseases, and malnutrition and dehydration have been linked to abnormal shedding or dysecdysis in snakes [38]. Inappropriate lighting, hygiene, space, temperature and humidity can all cause or exacerbate health conditions [34]. Despite the importance of these factors, only nine papers (9%) considered these two domains collectively. The fifth domain, which addresses mental experiences, was only mentioned in five articles (6%) in the published literature. As wild animals, Ball pythons may not have evolved coping mechanisms to adequately deal with all of the artificial stressors presented in a given captive environment [24,39,40], which in some cases could be at odds with the innate adaptations of the species. Consideration of mental stresses such as states of fear and frustration are necessary to consider a broad view of the animal’s experience and to ensure a holistic assessment of their welfare [9].

### 4.2. The Trade Chain

In particular, studies concerning welfare during the early stages of the trade chain are currently under-represented in the scientific literature (i.e., during wild capture, ranching and transportation). For wild caught and ranched snakes, crowded transport, unhygienic conditions, poor nutrition, poor environmental control, poor handling and aggression from co-occupants are just some of the factors that could cause suffering [25]. However, no welfare assessments of snake ranching can be found in the existing published scientific literature, and welfare during transportation appears to be a completely unrecorded issue [6]. Currently it is unclear whether there are any welfare provisions for the snakes prior to CITES and International Air Transport Association (IATA) regulations which come in to force during international border crossings. This is particularly concerning because the limited data available suggest mortality rates for reptiles in transit could be as high as 33% [6,41].

It is not only the welfare of wild caught and ranched snakes that requires further scientific attention. To date, very little formal research has been undertaken to understand the health and welfare consequences of selectively breeding wild sourced and captive reptiles [10]. Anecdotal reports of potential welfare issues linked to genetic manipulation can be found in hobbyist media (e.g., duckbills [42]), but only one study in the published scientific literature focuses on a single genetic disorder (wobble head syndrome) associated with a widely propagated phenotype of the Ball python [10]. For most recently established and ‘trendy’ morphs, there is not yet enough data to draw conclusions. However, as the ‘novelty’ of new morphs appears to have been prioritised over research into the health consequences of this inbreeding, it is possible that some new diseases and disorders resulting from selective breeding could be reported in the future.

Our review suggests there is insufficient literature to fully assess the welfare impacts of the trade in Ball pythons as exotic pets. This raises questions about the ethics of the industry as a whole; is it humane for us to continue trading and keeping animals without a comprehensive understanding of their experience? A group of key UK veterinary organisations proposed greater controls on the exotic pet business by only approving species for the pet trade where the animal’s needs have been fully researched and understood, and where there is reasonable evidence from published literature and professional experience that these needs can be met [43]. Warwick et al. [1] describe ‘positive lists’ a similar principle of using evidence-based methods to determine the suitability of species for trading and keeping. The proposal of positive lists has generally been well accepted among veterinarians and allied professionals and is in development processes across Europe and North America [1]. The information currently available for Ball pythons would likely be insufficient to justify their place in the exotic pet market, using the positive list principle.

### 4.3. Limitations

It is the nature of reviews such as this one that, as a result of the target words and journal databases used, some relevant articles may be missed. However, given our extensive yet balanced application of 26 different target words in relation to health, welfare and trade across three different journal databases, this review provides a comprehensive and useful representation of the existing scientific literature focused on the health and welfare of Ball pythons in the international pet trade. Furthermore, we recognise that information about the captive care of Ball pythons is also widely available in the hobbyist media and grey literature, and that our study could be considered limited by only including scientific literature in our analysis. However, the aim of our study was to assess the evidence base that advice and knowledge throughout the industry is based on. We believe that highlighting the scarcity of formal publications demonstrates that whatever other information exists for pet owners is likely based on a lack of scientific evidence. This raises questions about what premise local regulations and advisory standards of care are based on, and whether the ownership of Ball pythons as exotic pets can be considered as ‘responsible’. Furthermore, grey literature and hobbyist media can be subject to non-scientific ‘folklore husbandry’ [44,45], a term coined to describe methods of husbandry deemed ‘best practice’ without proper evaluation, justified by the notion that ‘it has always been done that way’ [44]. Therefore, by eliminating these types of sources we can be sure that the information gathered in our review is scientifically valid.

### 4.4. Recommendations

It is hoped that the evidence presented in this review can be used to improve welfare for captive Ball pythons. Acknowledging research gaps in the published scientific literature and focusing research towards previously neglected areas of the industry could help improve captive conditions and practices for snakes kept and traded for the exotic pet industry. More informative research would also be valuable to aid the implementation of policies that mitigate or minimise harm during the trade chain. In the case of the Ball python, this would particularly apply to regulations governing captive breeding practices in the USA and Europe, and wild capture and ranching methods in key source countries (i.e., Togo, Benin and Ghana). 

Although broader research could improve captive conditions for Ball pythons, it is important to consider that the only way to fully mitigate all of the potential negative animal welfare impacts for Ball pythons is to remove them from the pet trade all together. This would require changes in legislation in conjunction with education and awareness campaigns to inspire behavioural changes among exotic pet owners and breeders. Schuppli et al. [25] argued that the wide-spread popularity of keeping non-traditional pet species renders it impractical to try to end exotic pet ownership and trade. Nevertheless, popularity should not be considered justification for an industry to continue if it is associated with animal suffering. In the absence of removing Ball pythons from the pet trade all together, any improvements to conditions and practices would be advantageous to the millions of snakes living within the industry.

## 5. Conclusions

Our review highlights that research addressing Ball pythons in the exotic pet trade chain is limited within the published literature. Moreover, the sparse information that does exist is focused on a few specific facets, neglecting several animal welfare domains and key stages of the trade chain that supports this commercial enterprise. This paucity of information could hinder efforts to safeguard the welfare of the animals involved.

From the limited literature available it is already clear there are a multitude of welfare issues associated with different aspects of the trade chain. The lack of research addressing welfare throughout each stage, particularly during captive breeding, ranching and transport, is likely limiting our awareness of the negative consequences that the exotic pet industry can have on Ball pythons. More importantly, millions of animals are currently experiencing these welfare threats first-hand. Consequently, we recommend particular focus should be placed on the potential consequences of selective captive-breeding for ‘rare’ morphs and conditions provided for snakes prior to and during international transportation, where there is currently a dearth of knowledge. It is hoped this study demonstrates that our current understanding of welfare for Ball pythons traded as exotic pets is superficial and would benefit from a broader range of research throughout the industry.

## Supplementary Materials

The following are available online at https://www.mdpi.com/2076-2615/10/2/193/s1, Table S1—Definition of terms (behaviour); Table S2—Definition of terms (health); Table S3—Definition of terms (bacteria); Table S4—Definition of terms (parasite); Table S5—Definition of terms (virus); Table S6—Definition of terms (protozoa).

## Appendix A. Table of assigned categories for primary focus of papers used in this study and definitions

**Assigned Category**	**Definitions**
Welfare	Primarily focused on the broader animal welfare (non-clinical: stress, harm, etc.) impacts on Ball pythons in captivity for commercial, private or zoological purposes.
Conservation	Primarily focused on Ball python conservation, wild populations and their management, and/or species status.
Veterinary science	Primarily focused on the pathology, clinical symptoms or treatments and procedures relating to Ball pythons.
Trade	Primarily focused on the trade of Ball pythons, other than welfare and conservation impacts. For example, scope and scale of trade, economic impacts and legislation or regulation.

## Appendix B. Table of negative behaviours and physical health afflictions mentioned in each of the papers in the dataset

**Domain**	**Terms (taken directly from source papers)**
Behaviour	Abnormal posture [46]; Anorexia [46,47]; Disorientation [47]; head tremors [47]; Incoordination [47]; Lethargy [46]; Open-mouthed breathing [48]; Regurgitation [47]; Stargazing [47]
Health	Hyperglycaemia [49]; Anemia [49]; Azurophilia [49]; bacterial infection (unspecified) [46,50,51,52]; bilateral corneal opacity [8]; bilateral corneal ulceration [53]; bronchial epithelial hyperplasia [54]; cardiac malformations [55]; **caudal paralysis [46]; central nervous system disease [46,47]; corneal ulceration [54]; dermatitis [54]; **dysecdysis [53]; ectoparasite presence [56]; elevated creatine kinase activity [49]; esophagitis [48,57]; *facial cellulitis [58]; **facultative parthenogenesis [59]; focal dermatitis [46]; gastrointestinal tract diseases (unspecified) [60]; granulocytic meningomyelitis [46]; hamartoma [49]; hepatic lipidosis [54]; heteropenia [49]; moderate heterophilic and lymphocytic anterior uveitis [53]; heterophilic and lymphocytic keratoconjunctivitis with neovascularization and intralesional bacterial colonies [53]; histiocytic meningomyelitis [46]; hyperplasia [54]; Hyperuricemia [49]; leptospirosis [61]; lesions [54]; leukocytosis [49]; lymphocytic biliary dochitis [46]; lymphocytic encephalitis [46]; lymphocytic meningomyelitis [46]; lymphocytolysis [46]; lymphocytosis [49]; lympho proliferative disorder [47]; marked segmental degeneration [53]; mite infestation [49,62]; mucosal hemorrhages [48]; mucous metaplasia [54]; necrosis of stratum basale and spinosum [53]; necrotizing conjunctivitis [54]; nephritis nephrosis [54]; neuronal necrosis [46]; neuronophagia [46]; ocular disease [63]; opisthotonus [47]; oral bacteria [64]; pharyngitis [48,54]; pneumonia [46,47,48,54,57,65,66]; pulmonary hemorrhage [54]; renal lesions [67]; renal tubular degeneration [53]; respiratory disease [48,54,65]; salpingitis [54]; segmented epidermal erosion and ulceration [53]; sinusitis [48,54]; squamous cell carcinoma [49]; stomatitis [46,47,48,54]; superficial perivascular lymphocytic dermatitis [53]; subspectacular nematodiasis [68] tick parasatism [69,70,71,72]; tracheitis [48,54,57]; tracytoplasmic inclusion bodies [47]; under-developed ocular structures [73]; **wobble head syndrome [10]; bite wounds [49,52]; burns [49]; dermatologic lesions [49]; Inflammation [74,75]; skin incision made by researchers [76]

* Conditions that affect humans but not snakes ** Conditions that affect snakes but not humans. All other conditions can affect both humans and snakes.

## Appendix C. Table of the specific pathogens (categorized into bacteria, parasites, protozoa and viruses) mentioned in the papers in the dataset

**Pathogen**	**Species, serovars and diseases (taken directly from source papers)**
Bacteria	*Acinetobacter calcoaceticus* [64], *A. lwoffii* [77], *Aeromonas hydrophila* [64], *A. veronii* [77], *Anaplasma phagocytophilum* [78], *Bacteroides* spp. [64], *Bordetella hinzii* [65], chlamydophilosis [48], *Citrobacter freundii* [64,65,77], *Clostridium* spp. [64], *Elizabethkingia meningoseptica* [65], *Enterobacter cloacae* [64,65], *Enterococcus pallens* [77], *Escherichia coli* [51,64], *Klebsiella oxytoca* [49,65], *K. pneumoniae* [65], *Klebsiella* spp. [58], *Leptospira grippotyphosa* [61], *Lysobacter pythonis* [79], *Moraxella osloensis* [77], *Morganella morganii* [49,64], Mycoplasmosis [48], *Proteus vulgaris* [65], *Providencia rettgeri* [80], *Pseudomonas aeruginosa* [49,58,65,67], *P. floureszens* [65], *P. japonica* [77], *Pseudomonas* spp. [64], *Salmonella Muenchen* [65], *Salmonella Paratyphi* B [50,65], *Salmonella* spp. [49,64,65], *Salmonella* ssp. IIIb [65], *Salmonella* ssp. IV [65], *Serratia plymuthica* [77], *Staphylococcus* spp. [49,64,65], *Staphylococcus warneri* [77], *Stenotrophomonas maltophilia* [52,65,77], *Tsukamurella paurometabola* [77]
Parasites	*Amblyomma dissimile* [56], *A. exornatum* [69], *A. latum* [56,69,71,78], *A. rotundatum* [56], *Amblyomma* spp. [69,71,78], *A. transversal* [69,78], *Armillifer* spp. [81,82], *Eutrombicula cinnabaris* [56], *E. splendens* [56], *Geckobia hemidactyli* [56], *Hirstiella stamii* [56], *Ixodes scapularis* [70], *Linguatula* spp. [81], Nematoda spp. [48,83], *Ophionyssus natricis* [62,75], *Porocephalus* spp. [81], *Raillietiella* spp. [64], *Serpentirhabdias dubielzigi* [68,83], *Pentastomida* spp. [81]
Protozoa	*Hepatozoon ayorgbor* [84], *Hepatozoon* spp. [84,85,86], *Trypanosoma* cf. *varani* [87], *Trypanosoma* spp. [84,87]
Fungi	No species names reported
Viruses	Adenovirus [57,65], Barnivirus [48], Boid inclusion body disease [49,66], Chikungunya Virus [88], Circovirus [89], Ferlavirus [65], Filoviridae [54], Flaviviruses [57], Herpes viruses [57], Iridoviruses [57], Inclusion body disease [46,47,65,90,91], lymphocytic ganglioneuritis [46], Nidovirus [48,54,57], *Paramyxovirus* spp. [48,54,57,92,93], Reptarenaviruses [46,47], Reoviridae spp. [48,57], Retrovirus [91], Rhabdoviridae spp. [54], Torovirinae [48,54,57]

## References

[1] Warwick C., Steedman C., Jessop M., Arena P., Pilny A., Nicholas E. (2018). Exotic pet suitability: Understanding some problems and using a labeling system to aid animal welfare, environment, and consumer protection. J. Vet. Behav..

[2] Moorhouse T.P., Balaskas M., D’Cruze N.C., Macdonald D.W. (2016). Information Could Reduce Consumer Demand for Exotic Pets. Conserv. Lett..

[3] Robinson J.E., John F.A.V., Griffiths R.A., Roberts D.L. (2015). Captive reptile mortality rates in the home and implications for the wildlife trade. PLoS ONE.

[4] Auliya M. (2003). Hot Trade in Cool Creatures: A Review of the Live Reptile Trade in the European Union in the 1990s with a Focus on Germany.

[5] Auliya M., Altherr S., Ariano-Sánchez D., Baard E., Brown C., Brown R., Cantu C., Gentile G., Gildenhuys P., Henningheim E. (2016). Trade in live reptiles, its impact on wild populations, and the role of the European market. Biol. Conserv..

[6] Jensen T.J., Auliya M., Burgess N.D., Aust P.W., Pertoldi C., Strand J. (2019). Exploring the international trade in African snakes not listed on CITES: Highlighting the role of the internet and social media. Biodivers. Conserv..

[7] PFMA (2017). UK Pet Population Statistics. Pet Food Manufacturers’ Association..

[8] APPA (2017). National Pet Owners Survey 2017–2018.

[9] Warwick C., Arena P., Steedman C. (2019). Spatial considerations for captive snakes. J. Vet. Behav. Clin. Appl. Res..

[10] Rose M.P., Williams D.L. (2014). Neurological dysfunction in a Ball python (*Python regius*) colour morph and implications for welfare. J. Exot. Pet Med..

[11] World Animal Protection (2019). Exploiting Africa’s Wildlife—The ‘Big 5’ and ‘Little 5’. https://d31j74p4lpxrfp.cloudfront.net/sites/default/files/big_5_little_5_report.pdf.

[12] Paré J.A. An overview of pet reptile species and proper handling. Proceedings of the North American Veterinary Conference.

[13] Trape J.F., Mané Y. (2006). Savane et desert. Guide des serpents d’Afrique occidentale.

[14] Burghardt G.M. (2017). Keeping reptiles and amphibians as pets: Challenges and rewards. Vet. Rec..

[15] Broghammer S. (2018). Python Regius: Atlas of Colour Morphs, Keeping and Breeding.

[16] Bush E.R., Baker S.E., Macdonald D.W. (2014). Global trade in exotic pets 2006–2012. Conserv. Biol..

[17] Whitehead M.L. (2018). Factors contributing to poor welfare of pet reptiles. Testudo.

[18] Warwick C., Jessop M., Arena P., Pliny A., Nicholas E., Lambiris A. (2017). Future of keeping pet reptiles and amphibians: animal welfare and public health perspective. Veterinary Record..

[19] (2018). RSPCA Royal Python Care Sheet. https://www.rspca.org.uk/adviceandwelfare/pets/other/royalpython?gclid=EAIaIQobChMIo7r8mZP73AIV65XtCh1jvgcpEAAYASAAEgKlp_D_BwE.

[20] D’Cruze N., Paterson S., Green J., Megson D., Warwick C., Coulthard E., Norrey J., Auliya M., Carder G. Dropping the Ball: The welfare of Ball Pythons traded in the EU and North America. Animals.

[21] Baker S.E., Cain R., Van Kesteren F., Zommers Z.A., D’Cruze N., Macdonald D.W. (2013). Rough trade: Animal welfare in the global wildlife trade. BioScience.

[22] Arena P.C., Warwick C., Steedman C. (2014). Welfare and environmental implications of farmed sea turtles. J. Agric. Environ. Ethics.

[23] Warwick C., Lindley S., Steedman C. (2011). Signs of stress. Environ. Health News.

[24] Warwick C., Arena P., Lindley S., Jessop M., Steedman C. (2013). Assessing reptile welfare using behavioural criteria. InPractice.

[25] Schuppli C.A., Fraser D., Bacon H.J. (2014). Welfare of non-traditional pets. Rev. Sci. Tech..

[26] IUCN/SSC (2013). Guidelines for Reintroductions and Other Conservation Translocations, Version 1.0.

[27] Proctor H.S., Carder G., Cornish A. (2013). Searching for Animal Sentience: A Systematic Review of the Scientific Literature. Animals.

[28] Burghardt G.M. (2013). Environmental enrichment and cognitive complexity in reptiles and amphibians: Concepts, review, and implications for captive populations. Appl. Anim. Behav. Sci..

[29] De Vere A.J., Kuczaj S.A. (2016). Where are we in the study of animal emotions?. Wiley Interdiscip. Rev. Cogn. Sci..

[30] Benn A.L., McLelland D.J., Whittaker A.L. (2019). A Review of Welfare Assessment Methods in Reptiles, and Preliminary Application of the Welfare Quality^®^ Protocol to the Pygmy Blue-Tongue Skink, *Tiliqua adelaidensis*, Using Animal-Based Measures. Animals.

[31] Christoffel R.A., Lepczyk C.A. (2012). Representation of herpetofauna in wildlife research journals. J. Wildl. Manag..

[32] Warwick C. (1990). Reptilian ethology in captivity: Observations of some problems and an evaluation of their aetiology. Appl. Anim. Behav. Sci..

[33] Lambert H., Carder G., D’Cruze N. (2019). Given the Cold Shoulder: A Review of the Scientific Literature for Evidence of Reptile Sentience. Animals.

[34] Warwick C., Frye F.L., Murphy J.B. (2001). Health and Welfare of Captive Reptiles.

[35] Mellor D.J. (2017). Operational details of the five domains model and its key applications to the assessment and management of animal welfare. Animals.

[36] Hernandez-Divers S.J. (2001). Clinical aspects of reptile behavior. Vet. Clin. N. Am. Exot. Anim. Pract..

[37] Arena P., Steedman C., Warwick C. (2012). Amphibian and Reptile Pet Markets in the EU an Investigation and Assessment; Animal Protection Agency. https://www.apa.org.uk/pdfs/AmphibianAndReptilePetMarketsReport.pdf.

[38] Mutschmann F. (2008). Snake Diseases—Preventing and Recognizing Illness.

[39] Arena P.C., Warwick C., Warwick C., Frye F.L., Murphy J.B. (2004). Miscellaneous factors affecting health and welfare. Health and Welfare of Captive Reptiles.

[40] Warwick C., Warwick C., Frye F.L., Murphy J.B. (2004). Psychological and behavioural principles and problems. Health and Welfare of Captive Reptiles.

[41] Steinmetz M., Pütsch M., Bisschopinck T. (1998). Transport Mortality During the Import of Wild Caught Birds and Reptiles to Germany: An Investigation (Including a Study on Pre-Export-Conditions in the United Republic of Tanzania).

[42] Scherz M. Duckbill Mutation in Snakes. http://markscherz.tumblr.com/post/84960749508/what-is-the-duckbill-mutation.

[43] Wensley S., Dawson S., Stidworthy M., Soutar R. (2016). Welfare of exotic pets. Vet. Rec..

[44] Arbuckle K. (2013). Folklore husbandry and a philosophical model for the design of captive management regimes. Herpetol. Rev..

[45] Mendyk R.W., Berger M., Corbett S. (2018). Challenging folklore reptile husbandry in zoological parks. Zoo Animals: Husbandry, Welfare and Public Interactions.

[46] Stenglein M.D., Guzman D.S.M., Garcia V.E., Layton M.L., Hoon-Hanks L.L., Boback S.M., Keel M.K., Drazenovich T., Hawkins M.G., DeRisi J.L. (2017). Differential disease susceptibilities in experimentally reptarenavirus-infected boa constrictors and ball pythons. J. Virol..

[47] Chang L., Fu D., Stenglein M.D., Hernandez J.A., DeRisi J.L., Jacobson E.R. (2016). Detection and prevalence of boid inclusion body disease in collections of boas and pythons using immunological assays. Vet. J..

[48] Hoon-Hanks L.L., Layton M.L., Ossiboff R.J., Parker J.S., Dubovi E.J., Stenglein M.D. (2018). Respiratory disease in ball pythons (Python regius) experimentally infected with ball python nidovirus. Virology.

[49] White S.D., Bourdeau P., Bruet V., Kass P.H., Tell L., Hawkins M.G. (2011). Reptiles with dermatological lesions: a retrospective study of 301 cases at two university veterinary teaching hospitals (1992–2008). Vet. Dermatol..

[50] Krishnasamy V., Stevenson L., Koski L., Kellis M., Schroeder B., Sundararajan M., Ladd-Wilson S., Sampsel A., Mannell M., Classon A. (2018). Notes from the field: investigation of an outbreak of Salmonella Paratyphi B variant L (+) tartrate+(Java) associated with ball python exposure—United States, 2017. MMWR Morb. Mortal. Wkly. Rep..

[51] Larsen C.K., Skals M., Wang T., Cheema M.U., Leipziger J., Praetorius H.A. (2011). Python erythrocytes are resistant to α-hemolysin from Escherichia coli. J. Membr. Biol..

[52] Klinger C.J., Dengler B., Bauer T., Mueller R.S. (2018). Successful treatment of a necrotizing, multi-resistant bacterial pyoderma in a python with cold plasma therapy. Tierarztl. Prax. Ausg. K..

[53] Gardiner D.W., Baines F.M., Pandher K. (2009). Photodermatitis and photokeratoconjunctivitis in a ball python (Python regius) and a blue-tongue skink (Tiliqua spp.). J. Zoo Wildl. Med..

[54] Stenglein M.D., Jacobson E.R., Wozniak E.J., Wellehan J.F., Kincaid A., Gordon M., Porter B.F., Baumgartner W., Stahl S., Kelley K. (2014). Ball python nidovirus: a candidate etiologic agent for severe respiratory disease in Python regius. MBio.

[55] Jensen B., Wang T. (2009). Hemodynamic consequences of cardiac malformations in two juvenile ball pythons (Python regius). J. Zoo Wildl. Med..

[56] Corn J.L., Mertins J.W., Hanson B., Snow S. (2011). First reports of ectoparasites collected from wild-caught exotic reptiles in Florida. J. Med. Entomol..

[57] Uccellini L., Ossiboff R.J., De Matos R.E., Morrisey J.K., Petrosov A., Navarrete-Macias I., Jain K., Hicks A.L., Buckles E.L., Tokarz R. (2014). Identification of a novel nidovirus in an outbreak of fatal respiratory disease in ball pythons (Python regius). Virol. J..

[58] Bardi E., Vetere A., Aquaro V., Lubian E., Lauzi S., Ravasio G., Zani D.D., Manfredi M., Tecilla M., Roccabianca P. (2019). Use of Thrombocyte–Leukocyte-Rich Plasma in the Treatment of Chronic Oral Cavity Disorders in Reptiles: Two Case Reports. J. Exot. Pet Med..

[59] Booth W., Schuett G.W., Ridgway A., Buxton D.W., Castoe T.A., Bastone G., Bennett C., McMahan W. (2014). New insights on facultative parthenogenesis in pythons. Biol. J. Linn. Soc. Lond..

[60] Banzato T., Russo E., Finotti L., Zotti A. (2012). Development of a technique for contrast radiographic examination of the gastrointestinal tract in ball pythons (Python regius). Am. J. Vet. Res..

[61] Ajayi O.L., Antia R.E., Ojo O.E., Awoyomi O.J., Oyinlola L.A., Ojebiyi O.G. (2017). Prevalence and renal pathology of pathogenic Leptospira spp. in wildlife in Abeokuta, Ogun State, Nigeria. Onderstepoort. J. Vet. Res..

[62] Miranda R.J., Cleghorn J.E., Bermudez S.E., Perotti M.A. (2017). Occurrence of the mite Ophionyssus natricis (Acari: Macronyssidae) on captive snakes from Panama. Acarologia.

[63] Lauridsen H., Da Silva M.A.O., Hansen K., Jensen H.M., Warming M., Wang T., Pedersen M. (2014). Ultrasound imaging of the anterior section of the eye of five different snake species. BMC Vet. Res..

[64] Dipineto L., Russo T.P., Calabria M., De Rosa L., Capasso M., Menna L.F., Borrelli L., Fioretti A. (2014). Oral flora of P ython regius kept as pets. Lett. Appl. Microbiol..

[65] Schmidt V., Marschang R.E., Abbas M.D., Ball I., Szabo I., Helmuth R., Plenz B., Spergser J., Pees M. (2013). Detection of pathogens in Boidae and Pythonidae with and without respiratory disease. Vet. Rec..

[66] Wyss F., Schneiter M., Hetzel U., Keller S., Frenz M., Rička J., Hatt J.M. (2018). Investigation of the tracheal mucociliary clearance in snakes with and without boid inclusion body disease and lung pathology. J. Zoo Wildl. Med..

[67] Sala A., Di Ianni F., Pelizzone I., Bertocchi M., Santospirito D., Rogato F., Flisi S., Spadini C., Iemmi T., Moggia E. (2019). The prevalence of Pseudomonas aeruginosa and multidrug resistant Pseudomonas aeruginosa in healthy captive ophidian. PeerJ.

[68] Hausmann J.C., Mans C., Dreyfus J., Reavill D.R., Lucio-Forster A., Bowman D.D. (2015). Subspectacular nematodiasis caused by a novel Serpentirhabdias species in ball pythons (Python regius). J. Comp. Pathol..

[69] Mihalca A.D. (2015). Ticks imported to Europe with exotic reptiles. Vet. Parasitol..

[70] Yoder J.A., Rausch B.A., Jajack A.J., Tomko P.M., Gribbins K.M., Benoit J.B. (2013). Snakes produce kairomones that induce aggregation of unfed larval blacklegged ticks Ixodes scapularis (Acari: Ixodidae). Int. J. Acarol..

[71] Nowak M. (2010). Parasitisation and localisation of ticks [Acari: Ixodida] on exotic reptiles imported into Poland. Ann. Agr. Env. Med..

[72] Nowak M. (2010). The international trade in reptiles (Reptilia)—the cause of the transfer of exotic ticks (Acari: Ixodida) to Poland. Vet. Parasitol..

[73] Da Silva M.A.O., Bertelsen M.F., Wang T., Pedersen M., Lauridsen H., Heegaard S. (2015). Unilateral microphthalmia or anophthalmia in eight pythons (Pythonidae). Vet. Ophthalmol..

[74] Sadler R.A., Schumacher J.P., Rathore K., Newkirk K.M., Cole G., Seibert R., Cekanova M. (2016). Evaluation of the role of the cyclooxygenase signaling pathway during inflammation in skin and muscle tissues of ball pythons (Python regius). Am. J. Vet. Res..

[75] Schilliger L.H., Morel D., Bonwitt J.H., Marquis O. (2013). Cheyletus eruditus (Taurrus®): An effective candidate for the biological control of the snake mite (Ophionyssus natricis). J. Zoo Wildl. Med..

[76] Cole G.L., Lux C.N., Schumacher J.P., Seibert R.L., Sadler R.A., Henderson A.L., Odoi A., Newkirk K.M. (2015). Effect of laser treatment on first-intention incisional wound healing in ball pythons (Python regius). Am. J. Vet. Res..

[77] Zancolli G., Mahsberg D., Sickel W., Keller A. (2015). Reptiles as reservoirs of bacterial infections: real threat or methodological bias?. Microb. Ecol..

[78] Nowak M., Cieniuch S., Stańczak J., Siuda K. (2010). Detection of Anaplasmaphagocytophilum in Amblyomma flavomaculatum ticks (Acari: Ixodidae) collected from lizard Varanus exanthematicus imported to Poland. Exp. Appl. Acarol..

[79] Busse H.J., Huptas C., Baumgardt S., Loncaric I., Spergser J., Scherer S., Wenning M., Kämpfer P. (2019). Proposal of Lysobacter pythonis sp. nov. isolated from royal pythons (Python regius). Syst. Appl. Microbiol..

[80] Myers D.A., Wellehan J.F., Isaza R. (2009). Saccular lung cannulation in a ball python (Python regius) to treat a tracheal obstruction. J. Zoo Wildl. Med..

[81] Galecki R., Sokol R., Dudek A. (2016). Tongue worm (Pentastomida) infection in ball pythons (Python regius)-a case report. Ann. Parasitol..

[82] Ayinmode A.B., Adedokun A.O., Aina A., Taiwo V. (2010). The zoonotic implications of pentastomiasis in the royal python (Python regius). Ghana Med. J..

[83] Lucio-Forster A., Liotta J.L., Rishniw M., Bowman D.D. (2015). Serpentirhabdias dubielzigi n. sp.(Nematoda: Rhabdiasidae) from Captive-Bred Ball Pythons, Python regius (Serpentes: Pythonidae) in the United States. Comp. Parasitol..

[84] Halla U., Korbel R., Mutschmann F., Rinder M. (2014). Blood parasites in reptiles imported to Germany. Parasitol. Res..

[85] Tomé B., Maia J.P., Salvi D., Brito J.C., Carretero M.A., Perera A., Meimberg H., Harris D.J. (2014). Patterns of genetic diversity in Hepatozoon spp. infecting snakes from North Africa and the Mediterranean Basin. Syst. Parasitol..

[86] Haklová B., Majláthová V., Majláth I., Harris D.J., Petrilla V., Litschka-Koen T., Oros M., Peťko B. (2014). Phylogenetic relationship of Hepatozoon blood parasites found in snakes from Africa, America and Asia. Parasitology.

[87] Sato H., Takano A., Kawabata H., Une Y., Watanabe H., Mukhtar M.M. (2009). Trypanosoma cf. varani in an imported ball python (Python reginus) from Ghana. J. Parasitol..

[88] Bosco-Lauth A.M., Hartwig A.E., Bowen R.A. (2018). Reptiles and amphibians as potential reservoir hosts of Chikungunya virus. Am. J. Trop. Med. Hyg..

[89] Marton S., Ihász K., Lengyel G., Farkas S., Dán Á., Paulus P., Bányai K., Fehér E. (2015). Ubiquiter circovirus sequences raise challenges in laboratory diagnosis: the case of honey bee and bee mite, reptiles, and free-living amoebae. Acta Microbiol. Immunol. Hung..

[90] Chang L.W., Fu A., Wozniak E., Chow M., Duke D.G., Green L., Kelley K., Hernandez J.A., Jacobson E.R. (2013). Immunohistochemical detection of a unique protein within cells of snakes having inclusion body disease, a world-wide disease seen in members of the families Boidae and Pythonidae. PLoS ONE.

[91] Keilwerth M., Buehler I., Hoffmann R., Soliman H., El-Matbouli M. (2012). Inclusion Body Disease (IBD of Boids) a haematological, histological and electron microscopical study. Berl. Munch. Tierarztl. Wochenschr..

[92] Pees M., Schmidt V., Marschang R.E., Heckers K.O., Krautwald-Junghanns M.E. (2010). Prevalence of viral infections in captive collections of boid snakes in Germany. Vet. Rec..

[93] Marschang R.E., Papp T., Frost J.W. (2009). Comparison of paramyxovirus isolates from snakes, lizards and a tortoise. Virus Res..

