# Supplementary material for: Blind Trading: A Literature Review of Research Addressing the Welfare of Ball Pythons in the Exotic Pet Trade
Source: Animals (Basel). 2020 Jan 22;10(2):193. doi: 10.3390/ani10020193 (PMC7070511; doi:10.3390/ani10020193)
Supplement: Supplementary file 1 [file animals-10-00193-s001.pdf]

animals

Supplementary Files: Blind Trading: A Literature Review of Research Addressing the Welfare of Ball Pythons in the Exotic Pet Trade

Jennah Green [1,*], Emma Coulthard [2], David Megson [2], John Norrey [2], Laura Norrey [2], Jennifer K Rowntree [2], Jodie Bates [2], Becky Dharmpaul [1], Mark Auliya [3,4] and Neil D'Cruze [1,5]

This document is intended as support material for the manuscript 'Blind trading: A literature review of research addressing the welfare of Ball pythons in the exotic pet trade'.

We have provided definitions for each of the terms described in the appendices of the manuscript. Terms are divided into behaviour, health and pathogens (bacteria, parasite, protozoa and virus). Terms are exact terms used in the literature. All definitions were sourced from Merriam-Webster Medical dictionary, available at: https://www.merriam-webster.com/medical.

Table S1. Definition of terms (behaviour).

Behaviour	Definition
Abnormal posture	Not given
anorexia	Loss of appetite especially when prolonged
Disorientation	A usually transient state of confusion especially as to time, place, or identity often as a result of disease or drugs
Head tremors	Tremors: a trembling or shaking usually from physical weakness, emotional stress, or disease
Incoordination	Lack of coordination especially of muscular movements resulting from loss of voluntary control
Lethargy	Abnormal drowsiness or the quality or state of being lazy, sluggish, or indifferent
Open-mouthed breathing	Not given
Regurgitation	An act of regurgitating such as<br>a: the casting up of incompletely digested food (as by some birds in feeding their young)<br>b : the backward flow of blood through a defective heart valve
Stargazing	The quality or state of being absentminded

Table S2. Definition of terms (health).

Health	Definition
Hyperglycaemia	An excess of sugar in the blood
Anemia	Condition in which the blood is deficient in red blood cells, in hemoglobin, or in total volume
Azurophilia	No definition given
Bacterial infection (unspecified)	No definition
Bbilateral corneal opacity	Bilateral<br>: of, relating to, or affecting the right and left sides of the body or the right and left members of paired organs<br>Cornea<br>: the transparent part of the coat of the eyeball that covers the iris and pupil and admits light to the interior

	Opacity<br>: an opaque spot in a normally transparent structure (as the lens of the eye)
Bilateral corneal ulceration	Ulcer<br>1: a break in skin or mucous membrane with loss of surface tissue, disintegration and necrosis of epithelial tissue, and often pus<br>2: something that festers and corrupts like an open sore
Bronchial epithelial hyperplasia	Bronchial<br>: of or relating to the bronchi or their ramifications in the lungs<br>Bronchus<br>: either of the two primary divisions of the trachea that lead respectively into the right and the left lung<br>Epithelium<br>: a membranous cellular tissue that covers a free surface or lines a tube or cavity of an animal body and serves especially to enclose and protect the other parts of the body, to produce secretions and excretions, and to function in assimilation<br>Hyperplasia<br>: an abnormal or unusual increase in the elements composing a part (as cells composing a tissue)
Cardiac malformations	Cardiac (Entry 1 of 2)<br>a: of, relating to, situated near, or acting on the heart<br>b: of or relating to the cardia of the stomach<br>Malformation<br>: irregular, anomalous, abnormal, or faulty formation or structure
Caudal paralysis	Caudal<br>1: of, relating to, or being a tail<br>2: situated in or directed toward the hind part of the body<br>Paralysis<br>: complete or partial loss of function especially when involving the power of motion or of sensation in any part of the body
Central nervous system disease	Central nervous system<br>: the part of the nervous system which in vertebrates consists of the brain and spinal cord, to which sensory impulses are transmitted and from which motor impulses pass out, and which supervises and coordinates the activity of the entire nervous system<br>Disease<br>: an impairment of the normal state of the living animal or plant body or one of its parts that interrupts or modifies the performance of the vital functions, is typically manifested by distinguishing signs and symptoms, and is a response to environmental factors (as malnutrition, industrial hazards, or climate), to specific infective agents (as worms, bacteria, or viruses), to inherent defects of the organism (as genetic anomalies), or to combinations of these factors : sickness, illness
Corneal ulceration	Cornea<br>: the transparent part of the coat of the eyeball that covers the iris and pupil and admits light to the interior<br>Ulcer<br>1: a break in skin or mucous membrane with loss of surface tissue, disintegration and necrosis of epithelial tissue, and often pus

	2: something that festers and corrupts like an open sore
Dermatitis	Inflammation of the skin
Dysecdysis	No definition
Ectoparasite presence	A parasite that lives on the exterior of its host
Elevated creatine kinase activity	Creatine<br>: a white crystalline nitrogenous substance C4H9N3O2 found especially in vertebrate muscle either free or as phosphocreatine<br>Kinase<br>: any of various enzymes that catalyze the transfer of phosphate groups from a high-energy phosphate-containing molecule (as ATP or ADP) to a substrate
Esophagitis	Inflammation of the esophagus
Facial cellulitis	Diffuse and especially subcutaneous inflammation of connective tissue
Facultative parthenogenesis	Facultative<br>: taking place under some conditions but not under others<br>Parthenogenesis<br>: reproduction by development of an unfertilized usually female gamete that occurs especially among lower plants and invertebrate animals
Focal dermatitis	Inflammation of the skin
Gastrointestinal tract diseases (unspecified)	Gastrointestinal<br>: of, relating to, or affecting both stomach and intestine<br>Tract<br>: a system of body parts or organs that act together to perform some function<br>Disease<br>: an impairment of the normal state of the living animal or plant body or one of its parts that interrupts or modifies the performance of the vital functions, is typically manifested by distinguishing signs and symptoms, and is a response to environmental factors (as malnutrition, industrial hazards, or climate), to specific infective agents (as worms, bacteria, or viruses), to inherent defects of the organism (as genetic anomalies), or to combinations of these factors : sickness, illness
Granulocytic meningomyelitis	Granulocyte<br>: any of a group of white blood cells (as a basophil, eosinophil, or neutrophil) characterized by granule-containing cytoplasm and a usually lobed nucleus<br>Meningomyelitis<br>: inflammation of the spinal cord and its enveloping membranes
Hamartoma	A mass resembling a tumor that represents anomalous development of tissue natural to a part or organ rather than a true tumor
Hepatic lipidosis	Hepatic<br>: of, relating to, affecting, or associated with the liver<br>Lipidosis<br>: a disorder of fat metabolism especially involving the deposition of fat in an organ (as the liver or spleen)
Heteropenia	No definition
Moderate heterophilic and lymphocytic anterior uveitis	Heterophile<br>: of, relating to, or being an antibody circulating in blood serum

	that is reactive with antigen originating in a different species<br>Lymphocyte<br>: any of the colorless weakly motile cells that originate from stem cells and differentiate in lymphoid tissue (as of the thymus or bone marrow), that are the typical cellular elements of lymph, that include the cellular mediators of immunity, and that constitute 20 to 30 % of the white blood cells of normal human blood<br>Anterior<br>: relating to or situated near or toward the head or toward the part in headless animals most nearly corresponding to the head<br>Uveitis<br>: inflammation of the uvea
Heterophilic and lymphocytic keratoconjunctivitis with neovascularization and intralesional bacterial colonies	Heterophile<br>: of, relating to, or being an antibody circulating in blood serum that is reactive with antigen originating in a different species<br>Lymphocyte<br>: any of the colorless weakly motile cells that originate from stem cells and differentiate in lymphoid tissue (as of the thymus or bone marrow), that are the typical cellular elements of lymph, that include the cellular mediators of immunity, and that constitute 20 to 30 % of the white blood cells of normal human blood<br>Keratoconjunctivitis<br>: combined inflammation of the cornea and conjunctiva<br>Neovascularization<br>: vascularization especially in abnormal quantity (as in some conditions of the retina) or in abnormal tissue (as a tumor)<br>Intralesional<br>: introduced into or performed within a lesion<br>Bacterial<br>: of, relating to, or caused by bacteria<br>Colony<br>: a circumscribed mass of microorganisms usually growing in or on a solid medium
Histiocytic meningomyelitis	Histiocytic<br>: of, relating to, or containing macrophages<br>Meningomyelitis<br>: inflammation of the spinal cord and its enveloping membranes
Hyperplasia	An abnormal or unusual increase in the elements composing a part (as cells composing a tissue)
Hyperuricacidaemia	No definition
Leptospirosis	Any of several diseases of humans and domestic animals (as cattle and dogs) that are caused by infection with spirochetes of the genus Leptospira
Lesions	An abnormal change in structure of an organ or part due to injury or disease especially one that is circumscribed and well defined
Leukocytosis	An increase in the number of white blood cells in the circulating blood that occurs normally (as after meals) or abnormally (as in some infections)
Lymphocytic biliary dochitis	Lymphocyte<br>: any of the colorless weakly motile cells that originate from stem cells and differentiate in lymphoid tissue (as of the thymus or bone marrow), that are the typical cellular elements of lymph, that

	include the cellular mediators of immunity, and that constitute 20 to 30 % of the white blood cells of normal human blood<br>Biliary<br>: of, relating to, or conveying bile<br>No definition for dochitis
Lymphocytic encephalitis	Lymphocyte<br>: any of the colorless weakly motile cells that originate from stem cells and differentiate in lymphoid tissue (as of the thymus or bone marrow), that are the typical cellular elements of lymph, that include the cellular mediators of immunity, and that constitute 20 to 30 % of the white blood cells of normal human blood<br>Encephalitis<br>: inflammation of the brain that is caused especially by infection with a virus (such as herpes simplex, varicella zoster, or West Nile virus) or less commonly by bacterial or fungal infection or autoimmune reaction
Lymphocytic meningomyelitis	Lymphocyte<br>: any of the colorless weakly motile cells that originate from stem cells and differentiate in lymphoid tissue (as of the thymus or bone marrow), that are the typical cellular elements of lymph, that include the cellular mediators of immunity, and that constitute 20 to 30 % of the white blood cells of normal human blood<br>Meningomyelitis<br>: inflammation of the spinal cord and its enveloping membranes
Lymphocytolysis	No definition
Lymphocytosis	An increase in the number of lymphocytes in the blood usually associated with chronic infections or inflammations
Lympho proliferative disorder	Lympho-<br>: lymph : lymphatic tissue<br>Proliferative<br>: capable of or engaged in proliferation
Marked segmental degeneration	Segmental<br>a: of, relating to, or having the form of a segment<br>b: situated in, affecting, or performed on a segment<br>Degeneration<br>: progressive deterioration of physical characters from a level representing the norm of earlier generations or forms: regression of the morphology of a group or kind of organism toward a simpler less highly organized state
Mite infestation	any of numerous small to very minute arachnids of the order Acari that have a body without a constriction between the cephalothorax and abdomen, mandibles generally chelate or adapted for piercing, usually four pairs of short legs in the adult and but three in the young larvae, and often breathing organs in the form of tracheae and that include parasites of insects and vertebrates some of which are important disease vectors, parasites of plants in which they frequently cause gall formation, pests of various stored products, and completely innocuous free-living aquatic and terrestrial forms
Mucosal hemorrhages	Mucosa<br>: a membrane rich in mucous glands that lines body passages and cavities (as of the digestive, respiratory, and genitourinary tracts)

	which connect directly or indirectly with the exterior<br>Hemorrhage (Entry 1 of 2)<br>: a copious discharge of blood from the blood vessels
Mucous metaplasia	Mucous defined as above<br>Metaplasia<br>1: transformation of one tissue into another<br>metaplasia of cartilage into bone<br>2: abnormal replacement of cells of one type by cells of another
Necrosis of stratum basale and spinosum	Necrosis<br>: death of living tissue<br>Stratum basale<br>: the basal layer of the epidermis consisting of a single row of columnar or cuboidal epithelial cells that continually divide and replace the rest of the epidermis as it wears away<br>No definition for spinosum
Necrotizing conjunctivitis	Necrotizing<br>: causing, associated with, or undergoing necrosis<br>Conjunctivitis<br>: inflammation of the conjunctiva that is typically marked by pinkness or redness of the sclera and by itching, burning, irritation, discharge, or excessive tearing of the eye and that is typically caused by pathogenic microorganisms (such as bacteria or viruses), allergens, or irritants
Nephritis	Acute or chronic inflammation of the kidney affecting the structure (as of the glomerulus or parenchyma) and caused by infection, a degenerative process, or vascular disease
Nephrosis	A noninflammatory disease of the kidneys chiefly affecting function of the nephrons
Neuronal necrosis	Neurone<br>: one of the cells that constitute nervous tissue, that have the property of transmitting and receiving nerve impulses, and that are composed of somewhat reddish or grayish protoplasm with a large nucleus containing a conspicuous nucleolus, irregular cytoplasmic granules, and cytoplasmic processes which are highly differentiated frequently as multiple dendrites or usually as solitary axons and which conduct impulses toward and away from the cell body<br>Necrosis<br>: death of living tissue
Neuronophagia	Destruction of neurons by phagocytic cells
Ocular disease	Ocular<br>: of or relating to the eye<br>Disease definition as described previously
opisthotonos	a condition of spasm of the muscles of the back, causing the head and lower limbs to bend backward and the trunk to arch forward
Oral bacteria	Oral<br>: of, relating to, or involving the mouth<br>Bacteria<br>: a domain in the system of classification dividing all organisms into three major domains of life that includes the prokaryotes that are bacteria but not those that are archaebacteria or archaea

Pharyngitis	Inflammation of the pharynx (as from bacterial infection)
Pneumonia	An acute disease that is marked by inflammation of lung tissue accompanied by infiltration of alveoli and often bronchioles with white blood cells (as neutrophils) and fibrinous exudate, is characterized by fever, chills, cough, difficulty in breathing, fatigue, chest pain, and reduced lung expansion, and is typically caused by an infectious agent (as a bacterium, virus, or fungus)
Pulmonary haemorrhage	Pulmonary<br>: relating to, functioning like, associated with, or carried on by the lungs<br>Haemorrhage<br>: a copious discharge of blood from the blood vessels
Renal lesions	Renal<br>: relating to, involving, affecting, or located in the region of the kidney<br>Lesion<br>: an abnormal change in structure of an organ or part due to injury or disease especially one that is circumscribed and well defined
Renal tubular degeneration	Renal<br>: relating to, involving, affecting, or located in the region of the kidney<br>Tubular<br>1: having the form of or consisting of a tube<br>2: of, relating to, or sounding as if produced through a tube or tubule<br>Degeneration<br>1: intellectual or moral decline tending toward dissolution of character or integrity: a progressive worsening of personal adjustment<br>2a: progressive deterioration of physical characters from a level representing the norm of earlier generations or forms: regression of the morphology of a group or kind of organism toward a simpler less highly organized state<br>parasitism leads to degeneration<br>2b: deterioration of a tissue or an organ in which its vitality is diminished or its structure impaired
Respiratory disease	Respiratory<br>1: of or relating to respiration, respiratory function, respiratory diseases<br>2: serving for or functioning in respiration<br>Disease<br>: an impairment of the normal state of the living animal or plant body or one of its parts that interrupts or modifies the performance of the vital functions, is typically manifested by distinguishing signs and symptoms, and is a response to environmental factors (as malnutrition, industrial hazards, or climate), to specific infective agents (as worms, bacteria, or viruses), to inherent defects of the organism (as genetic anomalies), or to combinations of these factors: sickness, illness
Salpingitis	Inflammation of a fallopian or eustachian tube
Segmented epidermal erosion &	Epidermal

ulceration	: of, relating to, or arising from the epidermis<br>Erosion<br>1a: the superficial destruction of a surface area of tissue (as mucous membrane) by inflammation, ulceration, or trauma erosion of the uterine cervix<br>1b: progressive loss of the hard substance of a tooth<br>Ulceration<br>: the process of becoming ulcerated : the state of being ulcerated
Sinusitis	Inflammation of a sinus of the skull
Squamous cell carcinoma	Squamous<br>1a: covered with or consisting of scales<br>1b: of, relating to, or being a stratified epithelium that consists at least in its outer layers of small scalelike cells<br>Carcinoma<br>: a malignant tumor of epithelial origin
Stomatitis	Any of numerous inflammatory diseases of the mouth
Subspectacular nematodiasis	No definition for subspectacular<br>Nematodiasis<br>: infestation with or disease caused by nematode worms
Superficial perivascular lymphocytic dermatitis	Superficial<br>1: of, relating to, or located near the surface superficial blood vessels<br>2: lying on, not penetrating below, or affecting only the surface superficial wounds<br>Perivascular<br>: of, relating to, occurring in, or being the tissues surrounding a blood vessel<br>Lymphocyte<br>: any of the colorless weakly motile cells that originate from stem cells and differentiate in lymphoid tissue (as of the thymus or bone marrow), that are the typical cellular elements of lymph, that include the cellular mediators of immunity, and that constitute 20 to 30 % of the white blood cells of normal human blood<br>Dermatitis<br>: inflammation of the skin
Tick parasatism	Tick<br>1: any of numerous bloodsucking arachnids that constitute the acarine superfamily Ixodoidea, are much larger than the closely related mites, attach themselves to warm-blooded vertebrates to feed, include important vectors of various infectious diseases of humans and lower animals, and although the immature larva has but six legs, may be readily distinguished from an insect by the complete lack of external segmentation<br>2: any of various usually wingless parasitic dipteran flies (as the sheep ked)<br>Parasitism<br>: an intimate association between organisms of two or more kinds especially one in which a parasite obtains benefits from a host which it usually injures
Tracheitis	Inflammation of the trachea
Tracytoplasmic inclusion bodies	No definition for tracytoplasmic<br>Inclusion body

	: an inclusion, abnormal structure, or foreign cell within a cell specifically an intracellular body that is characteristic of some virus diseases and that is the site of virus multiplication
Under-developed ocular Structures	Of or relating to the eye<br>The ocular adnexa include the eyelids and the lacrimal glands
Wobble head syndrome	No definition
Bite wounds	Bite, transitive verb<br>:to seize especially with teeth or jaws so as to enter, grip, or wound
Burns	To injure or damage by exposure to fire, heat, or radiation
Dermatologic lesions	Dermatologic<br>: of or relating to dermatology<br>Dermatology<br>: a branch of medicine dealing with the skin and its structure, functions, and diseases<br>Lesion<br>: an abnormal change in structure of an organ or part due to injury or disease especially one that is circumscribed and well defined
Inflammation	A local response to cellular injury that is marked by capillary dilatation, leukocytic infiltration, redness, heat, pain, swelling, and often loss of function and that serves as a mechanism initiating the elimination of noxious agents and of damaged tissue
Skin incision	A cut or wound of body tissue made especially in surgery

Table S3. Definition of terms (bacteria).

Bacteria	Definition
Acinetobacter calcoaceticus	Acinetobacter (below)<br>Calcoacetius no definition
Acinetobacter lwoffii	Acinetobacter<br>1: a genus of nonmotile, gram-negative bacteria (family Moraxellaceae) that are short rods typically growing in pairs or in chains, that occur in soil and water, and that are associated with opportunistic infections especially of the skin, lungs, and urinary tract<br>2: a bacterium of the genus Acinetobacter<br>No definition for lwoffii
Aeromonas hydrophila	No definition
Aeromonas veronii	No definition
Anaplasma phagocytophilum	Anaplasma<br>: a genus of parasitic, gram-negative, nonmotile bacteria of the family Anaplasmataceae that are transmitted chiefly by ticks and biting flies and infect the red blood cells of humans and animals<br>No definition for phagocytophilum
Bacteroides spp.	A genus of gram-negative anaerobic bacteria that belong to the family Bacteroidaceae, that have rounded ends, produce no endospores and no pigment, and that occur usually in the normal intestinal flora
Bordetella hinzii	A genus of bacteria comprising very short gram-negative strictly aerobic coccuslike bacilli and including the causative agent (B. pertussis) of whooping cough
Chlamydophilosis	No definition
Citrobacter freundii	No definition

Clostridium spp.	A genus of saprophytic rod-shaped or spindle-shaped usually gram-positive bacteria (family Clostridiaceae) that are anaerobic or require very little free oxygen and are nearly cosmopolitan in soil, water, sewage, and animal and human intestines, that are very active biochemically comprising numerous fermenters of carbohydrates with vigorous production of acid and gas, many nitrogen-fixers, and others which rapidly putrefy proteins, and that include important pathogens
Elizabethkingia meningoseptica	No definition
Enterobacter cloacae	Enterobacter<br>: a genus of aerobic gram-negative bacteria of the family Enterobacteriaceae that produce acid and gas from many sugars (as dextrose and lactose), form acetoin, are widely distributed in nature (as in feces, soil, water, and the contents of human and animal intestines), and include some that may be pathogenic<br>Cloaca<br>: the common chamber into which the intestinal, urinary, and generative canals discharge especially in monotreme mammals, birds, reptiles, amphibians, and elasmobranch fishes
Enterococcus pallens	Enterococcus<br>: any of a genus (Enterococcus) of gram-positive bacteria that resemble streptococci and were formerly classified with them<br>especially a bacterium (E. faecalis) normally present in the intestine<br>No definition for pallens
Escherichia coli	Escherichia<br>: a genus of aerobic gram-negative rod-shaped bacteria of the family Enterobacteriaceae that form acid and gas on many carbohydrates (as dextrose and lactose) but no acetoin and that include occasionally pathogenic forms (as some strains of E. coli) normally present in the human intestine and other forms which typically occur in soil and water<br>Coli (Entry 1 of 2)<br>: of or relating to bacteria normally inhabiting the intestine or colon and especially to species of the genus Escherichia (as E. coli)
Klebsiella oxytoca	No definition for oxytoca
Klebsiella pneumoniae	No definition for pneumoniae
Klebsiella spp.	A genus of nonmotile gram-negative rod-shaped and frequently encapsulated bacteria of the family Enterobacteriaceae that include causative agents of respiratory and urinary infections
Leptospira grippotyphosa	Leptospira<br>: a genus of extremely slender aerobic spirochetes (family Leptospiraceae) that are free-living or parasitic in mammals and include a number of important pathogens (as L. icterohaemorrhagiae of Weil's disease or L. canicola of canicola fever)<br>No definition for grippotyphosa
Lysobacter pythonis	No definition
Moraxella osloensis	Moraxella<br>: a genus of short rod-shaped gram-negative bacteria that is placed in either of two families (Moraxellaceae or Neisseriaceae) and includes the causative agent (M. lacunata) of Morax-Axenfeld conjunctivitis<br>No definition for osloensis
Morganella morganii	No definition

Mycoplasmosis	No definition
Proteus spp.	A genus of aerobic gram-negative bacteria of the family Enterobacteriaceae that ferment glucose but not lactose and decompose urea, that are usually motile by means of peritrichous flagella, and that include saprophytes in decaying organic matter and a common causative agent (P. mirabilis) of urinary tract infections
Proteus vulgaris	No definition for vulgaris
Providencia rettgeri	No definition for rettgeri
Pseudomonas aeruginosa	No definition for aeruginosa
Pseudomonas floureszens	No definition for floureszens
Pseudomonas japonica	No definition for japonica
Pseudomonas spp.	A genus (the type of the family Pseudomonadaceae) comprising short rod-shaped motile gram-negative bacteria including some saprophytes, a few animal pathogens, and numerous important plant pathogens
Salmonella Muenchen	Salmonella<br>: a genus of aerobic gram-negative rod-shaped nonspore-forming usually motile bacteria of the family Enterobacteriaceae that grow well on artificial media and form acid and gas on many carbohydrates but not on lactose, sucrose, or salicin, that are pathogenic for humans and other warm-blooded animals, and that cause food poisoning, acute gastrointestinal inflammation, typhoid fever, and septicaemia<br>No definition for Muenchen
Salmonella Paratyphi B	Salmonella definition as above<br>No definition for Paratyphi
Salmonella spp.	Salmonella definition as above
Salmonella subspecies IIIb	Salmonella definition as above<br>No definition
Salmonella subspecies IV	Salmonella definition as above<br>No definition
Serratia plymuthica	Serratia<br>: a genus of aerobic saprophytic flagellated rod-shaped bacteria of the family Enterobacteriaceae that occur as rods, commonly produce a bright red pigment, and include one (S. marcescens) associated with some human opportunistic infections<br>No definition for plymuthica
Staphylococcus spp.	any bacterium of the genus Staphylococcus
Staphylococcus warneri	Staphylococcus<br>: a genus of nonmotile gram-positive spherical bacteria that is placed in either of two families (Staphylococcaceae or Micrococcaceae), contains forms occurring singly, in pairs or tetrads, or in irregular clusters, and includes causative agents of various diseases and disorders (as food poisoning, skin infections, and endocarditis)<br>No definition for warneri
Stenotrophomonas maltophilia	No definition
Tsukamurella paurometabola	No definition for Tsukamurella<br>Paurometabola, in some classifications<br>: a group comprising all insects that are paurometabolous

Table S4. Definition of terms (parasite).

Parasite	Definition
Amblyomma dissimile	Amblyomma<br>: a genus of ixodid ticks including the lone star tick (A. americanum) of the southern United States and the African bont tick (A. hebraeum)<br>No definition for dissimile
Amblyomma exornatum	Amblyomma definition same as above<br>No definition for exornatum
Amblyomma latum	Amblyomma definition same as above<br>No definition for latum
Amblyomma rotundatum	Amblyomma definition same as above<br>No definition for rotundatum
Amblyomma spp.	Amblyomma definition same as above
Amblyomma transversale	Amblyomma definition same as above<br>No definition for transversale
Armillifer spp.	No definition
Eutrombicula cinnabaris	Eutrombicula<br>: a genus of rather large mites (family Trombidiidae) that have the body clearly demarked into cephalothorax and abdomen, are free-living as adults, and have larvae which are typical chiggers<br>No definition for cinnabaris
Eutrombicula splendens	Eutrombicula<br>: a genus of rather large mites (family Trombidiidae) that have the body clearly demarked into cephalothorax and abdomen, are free-living as adults, and have larvae which are typical chiggers<br>No definition for splendens
Geckobia hemidactyli	No definition
Hirstiella stamii	No definition
Ixodes scapularis	Ixodes<br>: a widespread genus of ixodid ticks comprising chiefly bloodsucking parasites of humans and animals that may transmit pathogenic microorganisms and including one (I. holocyclus) of Australia that is known to cause severe allergic reactions<br>No definition for scapularis
Linguatula spp.	Linguatula<br>: a genus of tongue worms that includes a cosmopolitan parasite (L. serrata) of the nasal and respiratory passages of various canines, sheep and goats, the horse, and occasionally humans
Nematodes	Any of a phylum (Nematoda or Nemata) of elongated cylindrical worms parasitic in animals or plants or free-living in soil or water
Ophionyssus natricis	No definition
Porocephalus spp.	the type genus of the family Porocephalidae of tongue worms
Raillietiella spp.	No definition
Serpentirhabdias dubielzigi	No definition
Tongueworm (*Pentastomida spp.*)	Pentastomida<br>: any of a group of parasitic animals that are considered a class of the phylum Arthropoda or a separate phylum, that lack eyes, a circulatory system, and a respiratory system, that live as adults in the respiratory passages or body cavity

	of reptiles, birds, or mammals and undergo larval development in similar hosts, and that comprise the tongue worms
Undisclosed parasite spp.	N/A

Table S5. Definition of terms (protozoa).

Protozoa	Definition
Hepatozoon spp.	No definition given
Trypanosoma spp.	the type genus of the family Trypanosomatidae comprising kinetoplastid flagellates that as adults are elongated and somewhat spindle-shaped, have a posteriorly arising flagellum which passes forward at the margin of an undulating membrane and emerges near the anterior end of the body as a short free flagellum, and are parasitic in the blood or rarely the tissues of vertebrates, that following development in the digestive tract of a blood-sucking invertebrate and usually an insect pass ultimately to the mouthparts or salivary structures where they may be transmitted into a new vertebrate host bitten by the invertebrate host, and that are responsible for various serious diseases (as Chagas disease, dourine, nagana, sleeping sickness, and surra) of humans and domestic animals

Table S6. Definition of terms (virus).

Virus	Definition
Adenovirus	Any of a family (Adenoviridae) of double-stranded DNA viruses originally identified in human adenoid tissue, causing infections of the respiratory system, conjunctiva, and gastrointestinal tract, and including some capable of inducing malignant tumors in experimental animals
Barnivirus	No definition given
Boid inclusion body disease	No definittion
Chikungunya Virus	A febrile disease that resembles dengue, occurs especially in parts of Africa, India, and southeastern Asia, and is caused by a togavirus of the genus Alphavirus (species Chikungunya virus) transmitted by mosquitoes especially of the genus Aedes
Circovirus	No definition
Ferlavirus	No definition
Filoviridae	A family of single-stranded RNA viruses that infect vertebrates, that have a pleomorphic usually bacilliform or filamentous shape with a helical nucleocapsid and a lipoprotein envelope with glycoprotein projections, and that include the Ebola viruses and the Marburg virus
Flaviviruses	A genus of the family Flaviviridae of single-stranded RNA viruses that are transmitted by arthropod vectors and especially by ticks and mosquitoes and that include the causative agents of dengue, Japanese B encephalitis, Saint Louis encephalitis, West Nile fever, and yellow fever<br>2: any virus of the genus Flavivirus or the family Flaviviridae
Herpes viruses	Any of a family (Herpesviridae) of double-stranded DNA viruses that include the cytomegalovirus and Epstein-Barr virus and the causative agents of chicken pox, herpes simplex, Marek's disease, roseola infantum, and shingles
Iridoviruses	Any of a family (Iridoviridae) of double-stranded DNA viruses that contain an outer icosahedral capsid and that infect insects, frogs, and fish
Inclusion body disease	An inclusion, abnormal structure, or foreign cell within a cell

Lymphocytic ganglioneuritis	Definition of lymphocyte<br>: any of the colorless weakly motile cells originating from stem cells and differentiating in lymphoid tissue (as of the thymus or bone marrow) that are the typical cellular elements of lymph, include the cellular mediators of immunity, and constitute 20 to 30 percent of the white blood cells of normal human blood<br>No definition for ganglioneuritis
Nidovirus	No definition
Reptarenaviruses	No definition
Retrovirus	Any of a family (Retroviridae) of single-stranded RNA viruses that produce reverse transcriptase by means of which DNA is produced using their RNA as a template and incorporated into the genome of infected cells, that are often tumorigenic, and that include the lentiviruses (such as the HIVs) and the causative agent of Rous sarcoma
Rhabdoviridae	A family of single-stranded RNA viruses that are rod- or bullet-shaped, are found in plants and animals, and include the causative agents of rabies and vesicular stomatitis
Torovirinae	No definition
Paramyxovirus spp.	Any of a family (Paramyxoviridae) of single-stranded RNA viruses that include the parainfluenza viruses, the respiratory syncytial virus, and the causative agents of canine distemper, measles, mumps, Newcastle disease, and rinderpest
Reoviruses spp.	Any of a family (Reoviridae) of double-stranded RNA viruses that have a virion with icosahedral structural symmetry but may appear spherical, that have a capsid with one to three concentric protein layers, and that include many plant or animal pathogens (such as the rotaviruses and the causative agent of bluetongue)
Rhabdoviridae	A family of single-stranded RNA viruses that are rod- or bullet-shaped, are found in plants and animals, and include the causative agents of rabies and vesicular stomatitis
